# Metabolomic Profiling Delineates Stage‐Associated Metabolic Remodelling in Cardiovascular‐Kidney‐Metabolic Syndrome

**DOI:** 10.1002/dmrr.70230

**Published:** 2026-09-24

**Authors:** Xuemei Gong, Chunyang Li, Jing Chen, Yujiao Wang, Wenge Tang, Xuehui Zhang, Jianzhong Yin, Xing Zhao, Haopeng Yu, Ping Fu, Xiaoxi Zeng

**Affiliations:** ^1^ Department of Nephrology Institute of Kidney Diseases West China Hospital of Sichuan University Chengdu China; ^2^ West China Biomedical Big Data Center West China Hospital of Sichuan University Chengdu China; ^3^ Department of Nephrology Affiliated Hospital of Zunyi Medical University Zunyi China; ^4^ Chongqing Municipal Center for Disease Control and Prevention Chongqing China; ^5^ School of Public Health Kunming Medical University Kunming China; ^6^ West China School of Public Health and West China Fourth Hospital Sichuan University Chengdu China; ^7^ Health Promotion and Food Nutrition & Safety Key Laboratory of Sichuan Province Sichuan University Chengdu China

**Keywords:** biomarkers, cardiovascular‐kidney‐metabolic syndrome, metabolic remodelling, metabolomics

## Abstract

**Aims:**

Cardiovascular–kidney–metabolic (CKM) syndrome represents an integrated continuum of metabolic, kidney, and cardiovascular abnormalities. However, the stage‐specific metabolic heterogeneity underlying this clinical framework remains incompletely characterised.

**Materials and Methods:**

We performed plasma metabolomic profiling in 1374 participants from the China Multi‐Ethnic Cohort across CKM stages 0–4. Participants from Chengdu and Chongqing provinces comprised the discovery cohort (*n* = 969), whereas those from Yunnan province comprised the validation cohort (*n* = 405). Stage‐associated metabolites were identified using prespecified pairwise comparisons with OPLS‐DA and covariate‐adjusted limma analyses. Weighted correlation network analysis identified coordinated metabolic modules. Machine‐learning models were developed to evaluate discrimination of advanced CKM (stages 3–4) using metabolite signatures independent of conventional CKM‐defining variables.

**Results:**

Among 858 endogenous metabolites, 261 unique metabolites were associated with CKM stages, revealing distinct metabolic patterns from early to advanced CKM. Stage 1 was characterised by altered lipid‐ and bile acid‐related metabolites, stage 2 by broader lipid and amino‐acid remodelling, and stage 3 by additional carbohydrate, aromatic amino‐acid, secondary bile acid, host–microbial, and renal‐handling signals. Metabolic separation between stages 3 and 4 was comparatively weak. WGCNA identified a CKM‐associated turquoise module, with γ‐glutamylvaline as a hub metabolite. A model incorporating age, sex, and eight metabolites derived from the discovery cohort showed favourable performance for distinguishing advanced CKM from earlier stages and achieved an AUROC of 0.874 (95% CI, 0.802–0.931) in the validation cohort.

**Conclusions:**

CKM stages exhibit distinct and non‐linear metabolic signatures. A compact metabolomic panel independent of conventional CKM‐defining variables may provide complementary molecular information for advanced CKM phenotyping and future risk stratification.

## Graphical abstract

Using widely targeted metabolomics, we profiled 1374 participants across CKM stages 0–4 and identified 261 metabolites exhibiting distinct, non‐linear patterns of remodelling in lipid, carbohydrate, amino acid, and redox/energy metabolism. WGCNA further identified a 148‐metabolite turquoise module associated with CKM stage, with γ‐glutamylvaline emerging as a hub metabolite. Stage‐associated analyses and WGCNA identified 53 candidate metabolites, from which a stable eight‐metabolite panel was derived. This panel effectively discriminated advanced from non‐advanced CKM in the validation cohort (AUROC = 0.874), supporting its potential for complementary molecular phenotyping of CKM.

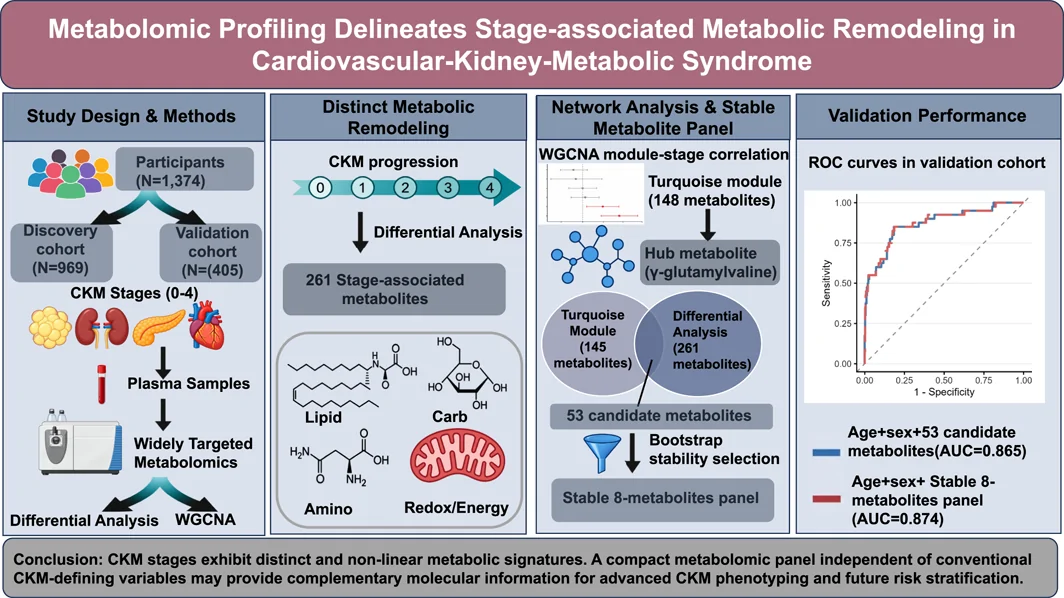

## 1 Introduction

Cardiovascular–kidney–metabolic (CKM) syndrome has emerged as an integrated framework for understanding the interdependence of metabolic disorders, chronic kidney disease (CKD), and cardiovascular disease (CVD) [1]. Population‐based studies suggest that a large proportion of adults have evidence of CKM syndrome [2], while approximately 10%–15% progress to advanced stages, which are associated with markedly increased risks of major adverse cardiorenal events compared with stage 0 [2, 3]. These findings highlight the need to understand the biological heterogeneity underlying the CKM stage continuum.

Metabolic dysregulation represents a potential biological link connecting the cardiovascular, kidney, and metabolic components of CKM. Previous metabolomic studies in obesity, diabetes, CKD, and CVD have identified abnormalities in amino acids (e.g., histidine, tryptophan, branched‐chain amino acids) [4, 5], lipids (e.g., lysophospholipid, acylcarnitines, sphingolipids) [6, 7, 8], and gut microbiota‐derived metabolites (e.g., short‐chain fatty acids, trimethylamine N‐oxide, indoxyl sulphate) [9, 10]. These alterations converge on pathways involved in lipid remodelling, amino‐acid metabolism, mitochondrial energy metabolism, inflammation, and oxidative stress [11, 12]. Large‐scale metabolomic profiling has further demonstrated associations between circulating metabolic signatures and cardiometabolic risk or incident CVD [11]. More recently, plasma metabolomics within the CKM framework revealed substantial interindividual metabolic variability associated with CKM risk [13]. However, existing studies have largely focused on individual disease components, broad disease comparisons, or overall CKM risk. Whether circulating metabolic patterns differ across the complete CKM stage 0–4 spectrum remains unclear.

Metabolomics provides a systems‐level assessment of biochemical activity and integrates genetic, environmental, dietary, and lifestyle influences, making it particularly suitable for investigating complex metabolic disorders [14]. For example, integrated lipid and amino acid profiles have outperformed conventional lipid markers in predicting cardiometabolic risk [15]. Beyond identifying individual biomarkers, metabolomic profiling may reveal coordinated metabolic networks, characterise subclinical biological states, and identify molecular signatures associated with disease progression. Nevertheless, a comprehensive, stage‐resolved characterisation of metabolic remodelling across the full CKM spectrum is lacking. However, metabolomic classification should not be viewed as a replacement for clinical staging. Rather, metabolomic profiling may provide complementary molecular information and capture biological heterogeneity that is not fully represented by conventional clinical criteria.

To address these knowledge gaps, we performed a cross‐sectional stage‐resolved plasma metabolomic analysis of 1374 participants spanning CKM stages 0–4 from the China Multi‐Ethnic Cohort (CMEC). We integrated stage‐specific differential metabolite analyses, metabolic network analysis, pathway enrichment, and machine‐learning approaches to characterise metabolic heterogeneity across CKM stages. This study aimed to identify stage‐associated metabolic signatures, define coordinated metabolic networks underlying CKM progression, and evaluate the potential complementary value of metabolomic information within the CKM framework.

## 2 Materials and Methods

### 2.1 Study Design and Population

This cross‐sectional study included 1374 Han Chinese participants from the CMEC metabolomics project recruited in Yunnan, Chongqing, and Sichuan, China. CMEC is a community‐based prospective cohort that enroled nearly 100,000 adults from five provinces in southwestern China between May 2018 and September 2019. Details of the cohort design, sampling strategy, and baseline characteristics have been described previously [16]. At baseline, sociodemographic characteristics, lifestyle factors, dietary information, and medical history were collected through face‐to‐face interviews using electronic questionnaires. Physical examinations and laboratory assessments were also performed, and fasting blood samples were collected. All participants provided written informed consent. The study was approved by the Medical Ethics Committee of Sichuan University and the local ethics committees at participating sites.

For the present analysis, participants from Chengdu and Chongqing study sites provinces comprised the discovery cohort (*n* = 969), whereas those from Yunnan province comprised the validation cohort (*n* = 405) (Supporting Information S1: Figure S1).

### 2.2 Ascertainment of CKM Syndrome

Based on a previous study [17], CKM syndrome stages were defined according to the AHA Presidential Advisory Statement [1], with operational adaptations based on the measurements available in CMEC. Briefly, the stages of CKM were defined as follows:

Stage 0: participants with BMI  < 23 kg/m^2^, waist circumference  < 80 cm for women and < 90 cm for men who did not meet the criteria for the other stages.

Stage 1: participants with BMI  ≥  23 kg/m^2^, waist circumference ≥ 80 cm for women and ≥ 90 cm for men, or glycated haemoglobin (HbA1c) of 5.7% to < 6.5% or a fasting blood glucose (FPG) of 100 mg/dL to < 126 mg/dL.

Stage 2: participants with metabolic risk factors (elevated fasting serum triglyceride levels, hypertension, diabetes, or metabolic syndrome) or moderate‐to high‐risk CKD according to Kidney Disease: Improving Global Outcomes criteria [18].

Stage 3: participants with a Predicting Risk of cardiovascular disease EVENTs (PREVENT) score indicating a 10‐year risk of CVD ≥ 20% [19], or very‐high‐risk CKD.

Stage 4: participants with self‐reported CVD (coronary heart disease, or stroke).

The detailed definitions of the above‐mentioned diseases used to define CKM in our study are listed in Table 1.

**TABLE 1:** Assessment of CKM syndrome stages.

CKM syndrome stages	Definition
Stage 0: No CKM risk factors	Participants with normal body mass index (BMI) (< 23 kg/m^2^), normal waist circumference (< 80 and < 90 cm for women and men, respectively) who did not meet criteria for the other stages.
Stage 1: Excess or dysfunctional adiposity	Individuals with an elevated BMI (≥ 23 kg/m^2^), an elevated waist circumference (≥ 80 and ≥ 90 cm for women and men, respectively), or prediabetes (defined as HbA1c of 5.7% to < 6.5% or FPG of 100 mg/dL to < 126 mg/dL).
Stage 2: Metabolic risk factors and moderate‐to‐high‐risk CKD	Participants with metabolic risk factors or moderate‐to‐high‐risk CKD^a^. Qualifying metabolic risk factors included hypertension^b^, diabetes^c^, metabolic syndrome^d^ or elevated fasting serum triglycerides (≥ 135 mg/dL)^e^.
Stage 3: Subclinical CVD in CKM, or very‐high‐risk CKD	Participants with a predicting 10‐year high cardiovascular risk^f^ or has a very‐high‐risk KDIGO CKD stages.
Stage 4: Clinical CVD in CKM	Self‐reported established cardiovascular disease (coronary heart disease, or stroke).

*Note:* The CKM definitions were based on the American Heart Association framework, with the following study‐specific operational adaptations. BMI and waist‐circumference cut‐off values followed the AHA criteria for Asian populations. Clinical CVD in CKM Stage 4 was defined using self‐reported established cardiovascular disease (coronary heart disease or stroke), rather than a physician‐adjudicated diagnosis. Missing values were observed for FPG (*n* = 55), BMI (*n* = 3), and HbA1c (*n* = 1). Because missingness was < 5% for all physical examination and laboratory measurements, missing values were imputed using multiple imputation.

Abbreviations: BMI, body mass index; CKD, chronic kidney disease; CKM, cardiovascular‐kidney‐metabolic; CVD, cardiovascular disease; eGFR, estimated glomerular filtration rate; FPG, fasting plasma glucose; HbA1c, glycated haemoglobin; HDL, high‐density lipoprotein; KDIGO, kidney disease: improving global outcomes; PREVENT, predicting risk of CVD EVENTs.

^a^ CKD risk classification was based on the Kidney Disease: Improving Global Outcomes cause‐glomerular filtration rate‐albuminuria framework. eGFR was calculated using the CKD‐EPI creatinine equation. Because urinary albumin‐to‐creatinine ratio was unavailable in CMEC, proteinuria was assessed using semiquantitative dipstick urinalysis. Dipstick proteinuria was categorised according to the approximate relationships reported by KDIGO: negative to trace for A1, trace to 1+ for A2, and ≥ 2+ for A3. These dipstick categories were treated as operational proxies rather than direct equivalents of quantitatively measured albuminuria categories [20].

^b^ Hypertension was defined by a previous diagnosis, current antihypertensive medication use, or mean systolic blood pressure ≥ 140 mmHg and/or mean diastolic blood pressure ≥ 90 mmHg.

^c^ Diabetes mellitus was defined by a self‐reported physician diagnosis, HbA1c ≥ 6.5%, or FPG ≥ 7.0 mmol/L.

^d^ Metabolic syndrome was defined as the presence of at least three of the following: waist circumference ≥ 80 cm in women or ≥ 90 cm in men; systolic blood pressure ≥ 130 mmHg or diastolic blood pressure ≥ 80 mmHg and/or antihypertensive medication use; FPG ≥ 100 mg/dL; HDL cholesterol < 50 mg/dL in women or < 40 mg/dL in men; and triglycerides ≥ 150 mg/dL.

^e^ Elevated triglycerides were defined by a history of hyperlipidaemia, lipid‐lowering medication use, or a triglyceride concentration ≥ 135 mg/dL.

^f^ High cardiovascular risk was defined as a predicted 10‐year CVD risk ≥ 20% using the AHA Predicting Risk of CVD EVENTs (PREVENT) equations. Values outside the supported PREVENT ranges were truncated to the nearest supported limit. Cardiac biomarkers and cardiovascular imaging were unavailable for identifying subclinical CVD.

### 2.3 Metabolomics Analysis

Plasma samples stored at −80°C were thawed in an ice–water bath. Aliquots (100 μL) were mixed with 300 μL methanol/acetonitrile/water (6:3:1, v/v/v) containing internal standards and 0.1 mmol/L butylated hydroxytoluene as an antioxidant. After vortexing and centrifugation to remove proteins, the extracts were lyophilised and reconstituted in water/methanol (4:1, v/v). Following centrifugation, the supernatants were subjected to LC–MS analysis. Quality‐control (QC) samples were prepared by pooling equal volumes of all study samples.

Metabolomic profiling was performed by Shanghai OE Biotech Co. Ltd. (Shanghai, China), following a plasma pseudotargeted metabolomics protocol based on ultra‐high‐performance liquid chromatography–tandem mass spectrometry [21]. Samples were analysed using an ExionLC/Nexera LC‐30A system coupled to a QTRAP 6500 mass spectrometer (AB Sciex).

Chromatographic separation was performed on a UPLC HSS T3 column (1.8 μm, 2.1 × 100 mm) at 45°C with a 5‐μL injection volume. The mobile phases were water containing 0.1% formic acid (A) and acetonitrile (B), delivered at 0.35 mL/min. The gradient increased from 5% B at 0–2 min to 30% at 4 min, 50% at 8 min, 80% at 10 min, and 100% at 14 min, followed by a 1‐min hold and re‐equilibration. Mass spectrometry was performed in positive‐ and negative‐ion modes using scheduled multiple‐reaction monitoring. Source parameters included a curtain gas pressure of 40 psi, medium collision gas, ion‐spray voltages of +5500 V and −4500 V, and Gas 1 and Gas 2 pressures of 50 and 55 psi, respectively.

### 2.4 Annotation of Metabolites and Data Processing

Raw data were processed using MRMPRO software [22] for peak extraction, alignment, identification, and area integration. Metabolic features were filtered by removing those with missing values (ion intensity = 0) in > 50% of samples; remaining zeros were imputed as half the minimum intensity across all samples. Features displaying a relative standard deviation (RSD) > 30% in QC samples were also excluded. After quality control, 1427 of the initially quantified 1428 metabolites were retained. Metabolites were annotated using the KEGG, HMDB, ChEBI, and LIPIDMAPS databases. Based on the relevant literature [23], endogenous metabolites were screened by excluding food‐derived and exogenous metabolites in accordance with HMDB 5.0. Ultimately, 858 plasma features were annotated as endogenous metabolites. All datasets were log‐transformed, sum‐centred, and auto‐scaled prior to downstream analyses.

### 2.5 Statistical Analyses

#### 2.5.1 Differential Metabolites Analyses

Cross‐sectional metabolic differences across CKM stages were evaluated in the discovery cohort using seven prespecified pairwise comparisons. Stages 1, 2, 3, and 4 were each compared with stage 0 as the reference, and three adjacent‐stage comparisons were additionally performed: stage 2 versus stage 1, stage 3 versus stage 2, and stage 4 versus stage 3. For each comparison, orthogonal partial least squares‐discriminant analysis (OPLS‐DA) was performed [24]. Variable importance in projection (VIP) scores were extracted for individual metabolites. Model performance was assessed using *R* ^2^ *Y*, *Q* ^2^, with robustness evaluated by 500 permutation tests.

In parallel, metabolite‐wise associations were evaluated using the limma framework with robust empirical Bayes moderation [25]. Models were adjusted for age, sex, smoking, drinking, physical activity, and educational attainment. Fold change (FC) was calculated from original intensities as the ratio of mean abundance in the comparison group to that in the reference group. Stage‐associated metabolites were defined as those meeting all three prespecified criteria: *p* < 0.05, FC > 1.20 or < 0.83, and VIP > 1 [26]. Volcano plots visualised the magnitude and direction of differences, and overlap plots summarised shared and comparison‐specific metabolites across contrasts.

#### 2.5.2 Weighted Gene Co‐Expression Network Analysis (WGCNA)

Weighted correlation network analysis was performed using WGCNA to identify coordinated metabolite modules associated with the CKM stage [27]. All 858 endogenous metabolites were included. A signed network was constructed using biweight midcorrelation with a maximum outlier proportion of 0.05. The soft‐thresholding power was selected using pickSoftThreshold as the lowest integer satisfying a signed scale‐free topology fit of *R* ^2^ ≥ 0.80, a negative slope, and mean connectivity ≥ 1, yielding a power of 9. The adjacency matrix was converted to a signed topological overlap matrix, followed by hierarchical clustering and dynamic tree cutting (deepSplit = 2; minimum module size = 20). Modules with highly correlated eigengenes were merged at a cut height of 0.25.

Module–stage associations were assessed using proportional‐odds logistic regression. CKM stage modelled as the ordinal outcome with adjustment for age, sex, smoking, drinking, physical activity, and educational attainment. Threshold‐specific binary logistic models were fitted to assess heterogeneity across stage boundaries and the proportional‐odds assumption. Module‐level *p* values were corrected using the Benjamini–Hochberg (BH) procedure, with False Discovery Rate (FDR) < 0.05 considered significant.

Module membership was quantified by the absolute eigengene correlation (|kME|), and metabolite significance was derived from adjusted metabolite–stage associations. Formal hub metabolites were required to belong to a CKM‐associated module with BH‐FDR < 0.05 and to have |kME| > 0.80 and an absolute adjusted metabolite–stage association > 0.20.

#### 2.5.3 Spearman Correlation Analysis and Functional Enrichment Analysis

Associations between metabolites and clinical parameters, including high‐density lipoprotein cholesterol (HDL‐C), total cholesterol (TC), triglycerides (TG), low‐density lipoprotein cholesterol (LDL‐C), BMI, FPG, HbA1c, blood urea nitrogen (BUN), and eGFR, were assessed using Spearman correlation. KEGG pathway enrichment analysis was performed separately for the stage‐associated metabolite sets and CKM‐associated WGCNA modules. Enrichment was assessed using Fisher's exact test, with *p* < 0.05 considered nominally significant.

#### 2.5.4 Development and Validation of a Classification Model for Advanced CKM Stages

Advanced CKM was defined as stages 3–4 and non‐advanced CKM as stages 0–2 [3]. We first developed a model incorporating age, sex, and metabolites that overlapped between the CKM‐associated turquoise module and the pooled set of metabolites identified across the seven stage‐specific comparisons.

We then developed a simplified model using a further selected subset of these candidate metabolites. Penalised logistic regression was prespecified as the primary algorithm, with ridge, elastic‐net, and LASSO penalties considered during tuning [28]. Random forest, XGBoost, and radial‐basis support vector machines were evaluated as secondary algorithms. Feature selection was restricted to the Discovery cohort. Elastic‐net/LASSO tuning used stratified cross‐validation and the one‐standard‐error rule, followed by 500 class‐stratified bootstrap replicates [29]. Metabolites with non‐zero coefficients in ≥ 60% of replicates were retained. Class imbalance was addressed using inverse‐frequency weighting without oversampling or synthetic observations. All preprocessing parameters were estimated exclusively within the corresponding training data.

To reduce direct incorporation bias, BMI, blood pressure, glycaemic indices, lipid measures, and eGFR were excluded because they contributed to the CKM‐stage assignment. Final models were evaluated in the validation cohort, and model performance was assessed using AUROC, PR‐AUC, sensitivity, specificity, positive and negative predictive values, balanced accuracy, Brier score, and calibration intercept and slope. Because advanced CKM was relatively uncommon, PR‐AUC was additionally reported to complement AUROC [30]. Clinical utility was assessed using decision‐curve analysis across threshold probabilities of 0.01–0.40 [31]. AUROCs from paired models were compared using DeLong's nonparametric test [32].

#### 2.5.5 Stratified and Sensitivity Analyses

To assess the robustness of the stage‐specific findings, we performed sensitivity and stratified analyses. Stage‐specific analyses were repeated after excluding participants whose CKM stage changed when CKD criteria were omitted and, separately, after excluding stage 4 participants. Scaling parameters were re‐estimated within each sensitivity dataset, and overlap and directional concordance with the primary results were quantified. The seven stage comparisons were also examined in sex‐ and age‐stratified analyses (< 60 and ≥ 60 years).

Analyses were conducted in R version 4.6.1 (R Foundation for Statistical Computing, Vienna, Austria). Two‐sided *p* < 0.05 was used unless otherwise specified.

## 3 Results

### 3.1 Cohort Construction and Metabolomics Profiling

A total of 1374 participants with available plasma metabolomic profiles and complete CKM staging information were included. Overall, 175, 142, 928, 75, and 54 participants were classified as CKM stages 0–4, respectively. Based on monitoring sites, 969 participants were assigned to the discovery cohort and 405 to the validation cohort. In the discovery cohort, 120 (12.4%), 107 (11.0%), 653 (67.4%), 50 (5.2%), and 39 (4.0%) participants were classified as stages 0–4, respectively; the corresponding numbers in the validation cohort were 55 (13.6%), 35 (8.6%), 275 (67.9%), 25 (6.2%), and 15 (3.7%).

**FIGURE 1:**
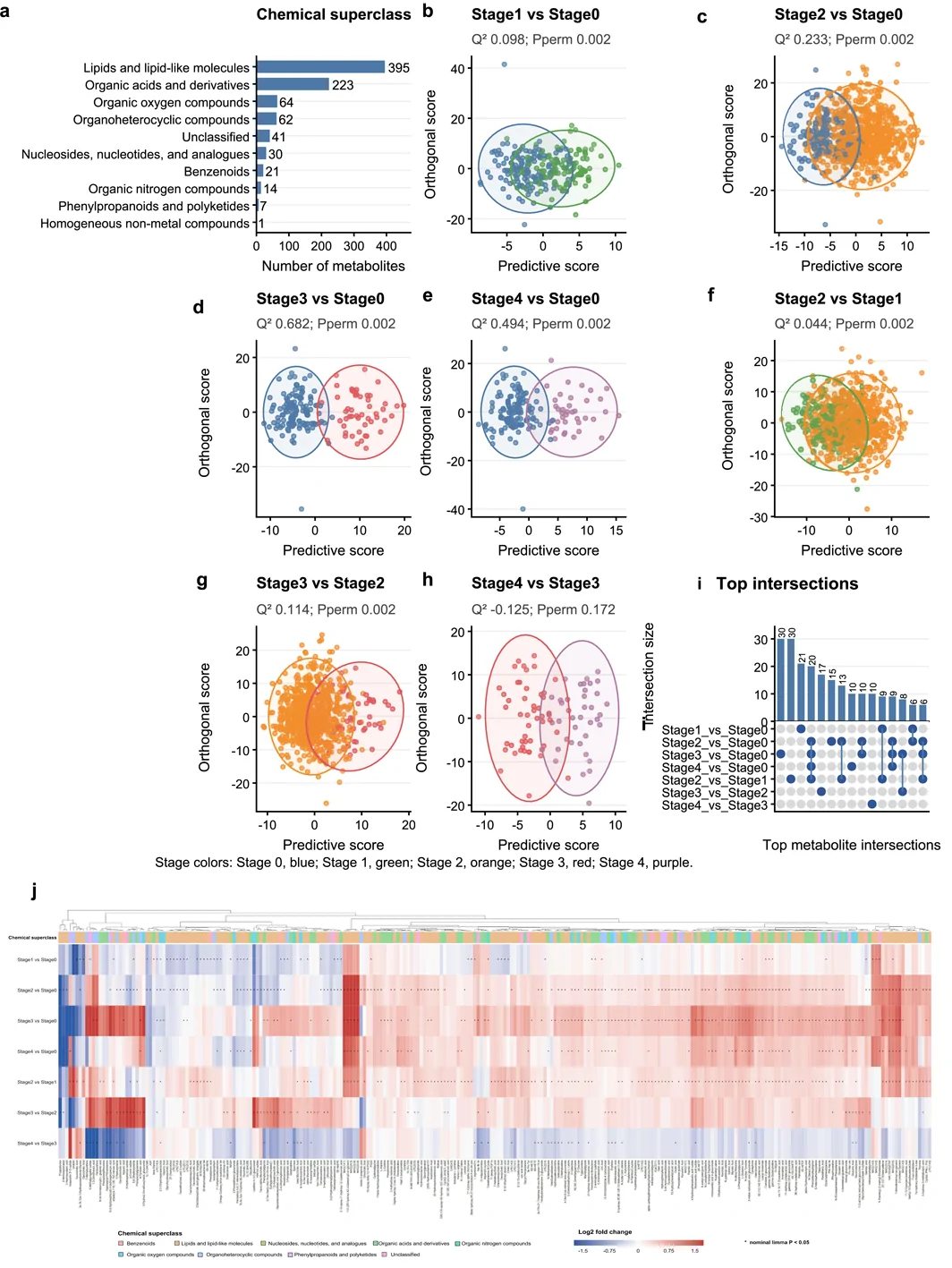
Stage‐associated metabolic remodelling across CKM stages. (a) Distribution of the 858 endogenous metabolites across chemical superclasses; (b) OPLS‐DA score plot comparing Stage 1 with Stage 0; (c) OPLS‐DA score plot comparing Stage 2 with Stage 0; (d) OPLS‐DA score plot comparing Stage 3 with Stage 0; (e) OPLS‐DA score plot comparing Stage 4 with Stage 0; (f) OPLS‐DA score plot comparing Stage 2 with Stage 1; (g) OPLS‐DA score plot comparing Stage 3 with Stage 2; (h) OPLS‐DA score plot comparing Stage 4 with Stage 3; (i) UpSet plot showing the leading intersections of differential metabolites across the seven prespecified CKM‐stage comparisons; (j) Heatmap showing log_2_ fold changes for the union of 261 differentially abundant metabolites across the seven comparisons. Red indicates higher abundance and blue indicates lower abundance in the target stage. Asterisks indicate nominal limma *p* < 0.05. The upper annotation denotes the chemical superclass of each metabolite. OPLS‐DA, orthogonal partial least‐squares discriminant analysis.

Median age was 51 years (IQR, 44–60) in the discovery cohort and 50 years (IQR, 43–61) in the validation cohort. Clinical characteristics varied across CKM stages, including age, adiposity, blood pressure, lipid and glycaemic profiles, kidney function, comorbidities, and medication use (Supporting Information S2: Table S1). After quality control and exclusion of food‐derived and other exogenous compounds, 858 endogenous metabolites were retained. Fatty acyls, steroids and steroid derivatives, and carboxylic acids and derivatives represented major chemical classes (Figure 1a).

**FIGURE 2:**
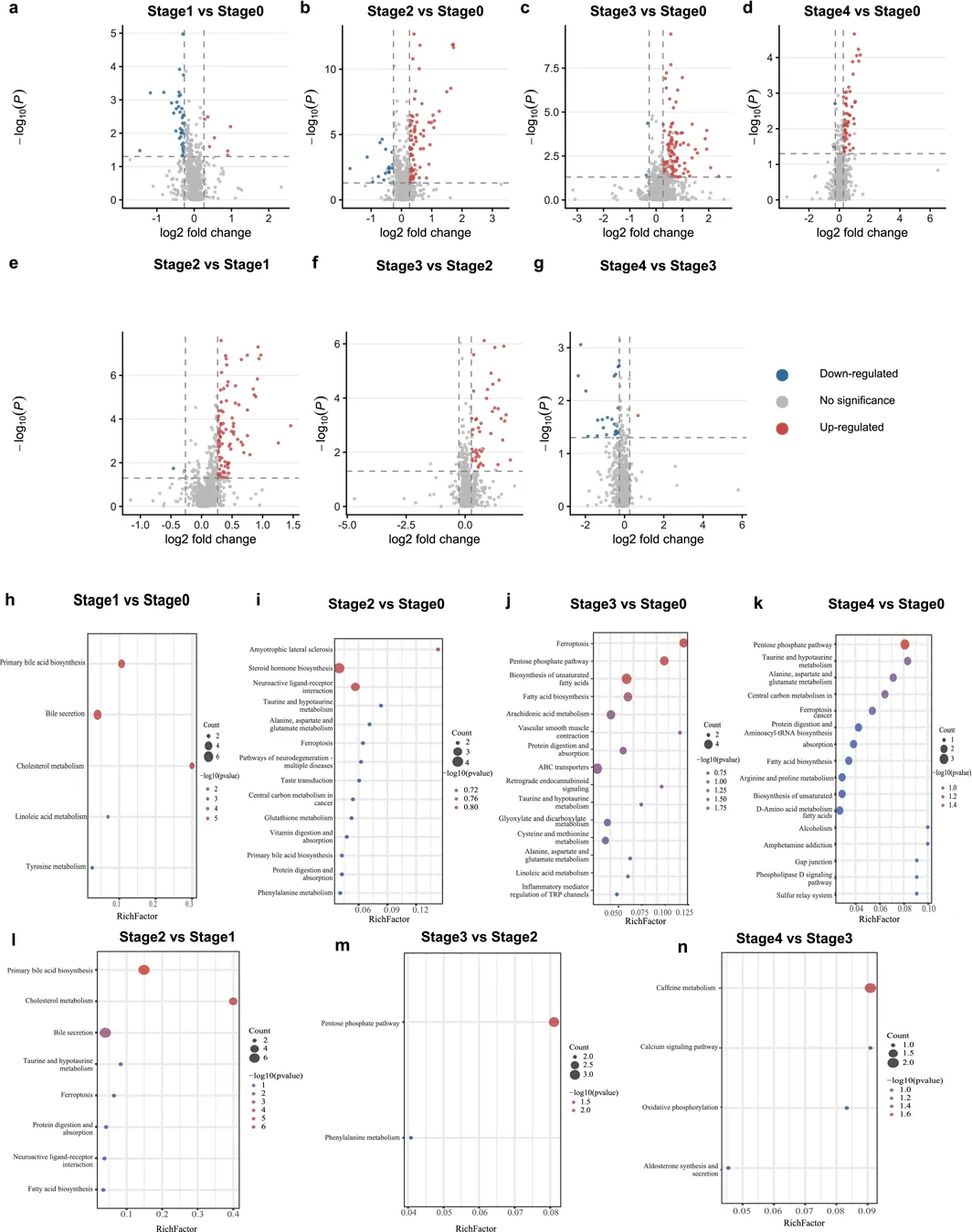
Stage‐associated metabolites and KEGG pathway enrichment across CKM stages. (a) Volcano plot of differential metabolites between Stage 1 and Stage 0; (b) Volcano plot of differential metabolites between Stage 2 and Stage 0; (c) Volcano plot of differential metabolites between Stage 3 and Stage 0; (d) Volcano plot of differential metabolites between Stage 4 and Stage 0; (e) Volcano plot of differential metabolites between Stage 2 and Stage 1; (f) Volcano plot of differential metabolites between Stage 3 and Stage 2; (g) Volcano plot of differential metabolites between Stage 4 and Stage 3; For (a–g), red and blue denote metabolites with higher and lower abundances, respectively, that met all three criteria: fold change > 1.2 or < 1/1.2, VIP > 1, and *p* < 0.05. Grey denotes metabolites that did not meet these criteria. (h) KEGG over‐representation analysis of differential metabolites identified between Stage 1 and Stage 0; (i) KEGG over‐representation analysis of differential metabolites identified between Stage 2 and Stage 0; (j) KEGG over‐representation analysis of differential metabolites identified between Stage 3 and Stage 0; (k) KEGG over‐representation analysis of differential metabolites identified between Stage 4 and Stage 0; (l) KEGG over‐representation analysis of differential metabolites identified between Stage 2 and Stage 1; (m) KEGG over‐representation analysis of differential metabolites identified between Stage 3 and Stage 2; (n) KEGG over‐representation analysis of differential metabolites identified between Stage 4 and Stage 3; For (h–n), pathways with enrichment *p* < 0.05 are displayed. Point size represents the number of mapped KEGG compounds, and colour represents −log_10_(*p*). RichFactor is the number of mapped differential‐metabolite compounds divided by the number of background compounds annotated to the pathway. FC, fold change; KEGG, Kyoto Encyclopaedia of Genes and Genomes; VIP, variable importance in projection.

### 3.2 Distinct and Non‐Linear Metabolic Profiles Across CKM Stages

Stage‐associated metabolic differences were characterised in the discovery cohort using seven prespecified comparisons. OPLS‐DA demonstrated differential metabolic separation across CKM stages (Figure 1b–h). The strongest separation was observed for stage 3 versus stage 0 (*R* ^2^ *Y* = 0.806, *Q* ^2^ = 0.682), followed by stage 4 versus stage 0 (*R* ^2^ *Y* = 0.737, *Q* ^2^ = 0.494) and stage 2 versus stage 0 (*R* ^2^ *Y* = 0.325, *Q* ^2^ = 0.233). In contrast, stage 4 versus stage 3 showed limited separation (*Q* ^2^ = −0.125), with neither *R* ^2^ *Y* nor *Q* ^2^ supported by permutation testing (*p* = 0.293 and 0.172, respectively) (Figure 1).

Across the seven comparisons, 261 unique stage‐associated metabolites were identified (Figures 1i,j and 2a–g; Supporting Information S2: Tables S2–S8). Compared with stage 0, 44, 96, 108, and 57 metabolites were identified for stages 1, 2, 3, and 4, respectively. Adjacent‐stage analyses identified 91 metabolites for stage 2 versus stage 1, 45 for stage 3 versus stage 2, and 28 for stage 4 versus stage 3. Stage 1 showed a predominantly decreased metabolic pattern, with 37 of 44 metabolites reduced (Figure 2a, Supporting Information S2: Table S2). Differential metabolites mainly included lysophospholipids, oxidised fatty‐acid derivatives, and bile acid‐related metabolites, such as LPE (20:2), tetranorprostanedioic acid, 9 (10)‐EpOME, glycocholic acid, LPC (20:1), and 12,13‐DHOME. Pathway enrichment highlighted primary bile acid biosynthesis, bile secretion, cholesterol metabolism, linoleic acid metabolism, and tyrosine metabolism (Figure 2h).

**FIGURE 3:**
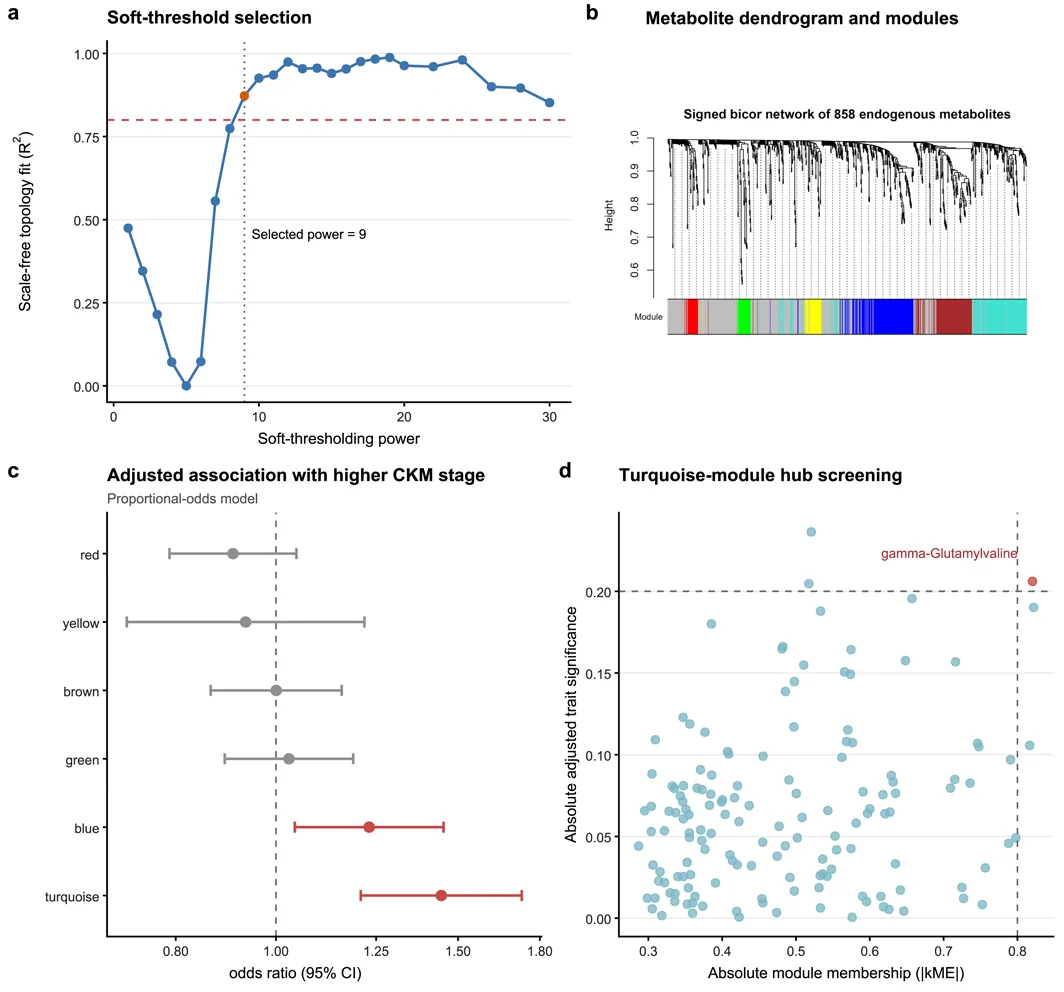
WGCNA‐derived metabolite modules associated with the CKM stage. (a) Scale‐free topology fit across candidate soft‐thresholding powers. Power 9 was selected after the scale‐free topology fit exceeded the prespecified *R* ^2^ threshold of 0.80. (b) Hierarchical clustering dendrogram and merged WGCNA module assignments for all 858 endogenous metabolites in the Discovery cohort. The network was constructed using signed biweight midcorrelations. (c) Adjusted proportional‐odds associations between module eigengenes and higher CKM stage. Points represent odds ratios, and horizontal lines represent 95% confidence intervals. Red indicates BH‐FDR < 0.05. Models were adjusted for age, sex, smoking, alcohol consumption, physical activity, and education. (d) Relationship between absolute module membership, |kME|, and absolute adjusted CKM‐stage trait significance within the turquoise module. Dashed lines indicate |kME| = 0.80 and trait significance = 0.20. Gamma‐glutamylvaline is highlighted in red. WGCNA, weighted gene co‐expression network analysis.

Stage 2 exhibited a broader metabolic alteration. Compared with stage 0, 79 of 96 differential metabolites were increased, including γ‐glutamylvaline, L‐glutamic acid, MAG(18:1), MAG(20:3), γ‐glutamylleucine, and N‐lactoyltyrosine (Figure 2b, Supporting Information S2: Table S3). Stage 2 versus stage 1 comparison showed a similar directional pattern, with 89 of 91 metabolites increased, particularly monoacylglycerols, fatty acids, and N‐acyl/lactoyl amino acids (Figure 2e, Supporting Information S2: Table S6). Corresponding pathways included bile acid and cholesterol metabolism, fatty‐acid biosynthesis, glutathione metabolism, taurine and hypotaurine metabolism, ferroptosis, and amino‐acid‐related pathways (Figure 2i,l).

Stage 3 demonstrated the largest number of differential metabolites. Compared with stage 0, 106 of 108 metabolites were increased, including D‐glucose, D‐galactose, N‐lactoyl amino acids, and γ‐glutamylvaline (Figure 2c, Supporting Information S2: Table S4). All 45 metabolites identified in stage 3 versus stage 2 were also increased, including phenylacetylglycine, phenylacetylglutamine, p‐cresol sulphate, secondary bile acids, and 4‐pyridoxic acid (Figure 2f, Supporting Information S2: Table S7). Enriched pathways included the pentose phosphate pathway, phenylalanine metabolism, ferroptosis, fatty‐acid metabolism, and arachidonic acid metabolism (Figure 2j,m).

Stage 4 remained distinct from stage 0, with 55 of 57 differential metabolites increasing. Representative metabolites included monoacylglycerols, 4‐pyridoxic acid, and L‐alanine (Figure 2d, Supporting Information S2: Table S5). Enriched pathways included central carbon metabolism, taurine and hypotaurine metabolism, amino‐acid metabolism, and fatty‐acid biosynthesis (Figure 2k). However, stage 4 versus stage 3 showed a weaker and qualitatively different pattern, with 27 of 28 metabolites decreased (Figure 2g, Supporting Information S2: Table S8) and limited pathway enrichment involving caffeine metabolism, calcium signalling, oxidative phosphorylation, and aldosterone synthesis and secretion (Figure 2n).

### 3.3 Stage‐Associated Metabolites Show Distinct Correlations With Clinical Characteristics

Spearman correlation analysis revealed stage‐specific associations between metabolites and clinical characteristics (Supporting Information S2: Table S9; Supporting Information S1: Figures S2–S8). Correlations were generally modest for stage 1 versus stage 0, with 12,13‐DHOME showing the strongest association with eGFR (*ρ* = 0.345; Supporting Information S1: Figure S2).

Stage 2 showed prominent associations with lipid‐related traits. In stage 2 versus stage 0, 1‐O‐ (2R‐hydroxy‐4Z‐octadecenyl)‐sn‐glycerol, MAG (18:1), and MAG (20:3) correlated strongly with triglycerides (*ρ* = 0.610, 0.605, and 0.602, respectively; Supporting Information S1: Figure S3). Similar associations were observed in stage 2 versus stage 1 (*ρ* = 0.555, 0.553, and 0.542, respectively; Supporting Information S1: Figure S4). L‐glutamic acid was positively correlated with BMI (*ρ* = 0.452) and FPG (*ρ* = 0.313), whereas γ‐glutamylvaline was inversely correlated with HDL‐C (*ρ* = −0.406).

Stage 3‐associated metabolites showed broader correlations involving glycaemic and kidney‐related traits. MAG (20:3) correlated with triglycerides (*ρ* = 0.623), 3‐indole carboxylic acid glucuronide inversely correlated with eGFR (*ρ* = −0.619), D‐galactose correlated with FPG (*ρ* = 0.574), and 3‐phenylpropionylglycine correlated with HbA1c (*ρ* = 0.528) (Supporting Information S1: Figure S5). Similar associations were observed for stage 3 versus stage 2, including correlations of D‐glucose with FPG (*ρ* = 0.552) and HbA1c (*ρ* = 0.457), and 4‐pyridoxic acid with eGFR (*ρ* = −0.284). (Supporting Information S1: Figure S6).

Stage 4 retained associations with lipid, glycaemic, and kidney‐related traits. MAG (18:1) and MAG (20:3) correlated positively with triglycerides (*ρ* = 0.556 and 0.543), γ‐glutamylvaline correlated with BUN (*ρ* = 0.488), and 3‐indole carboxylic acid glucuronide correlated inversely with eGFR (*ρ* = −0.504) (Supporting Information S1: Figure S7). In stage 4 versus stage 3, D‐glucose showed particularly strong correlations with FPG (*ρ* = 0.747) and HbA1c (*ρ* = 0.671) (Supporting Information S1: Figure S8).

### 3.4 WGCNA Identifies a CKM‐Associated Co‐Metabolic Module and γ‐Glutamylvaline as a Hub Metabolite

WGCNA of all 858 endogenous metabolites identified six non‐grey co‐metabolic modules in the discovery cohort (Figure 3a,b). After adjustment for clinical covariates, the turquoise module exhibited the strongest association with CKM stage among all modules. (OR = 1.45, 95% CI, 1.21–1.73; FDR = 3.26 × 10^−4^) (Figure 3c).

**FIGURE 4:**
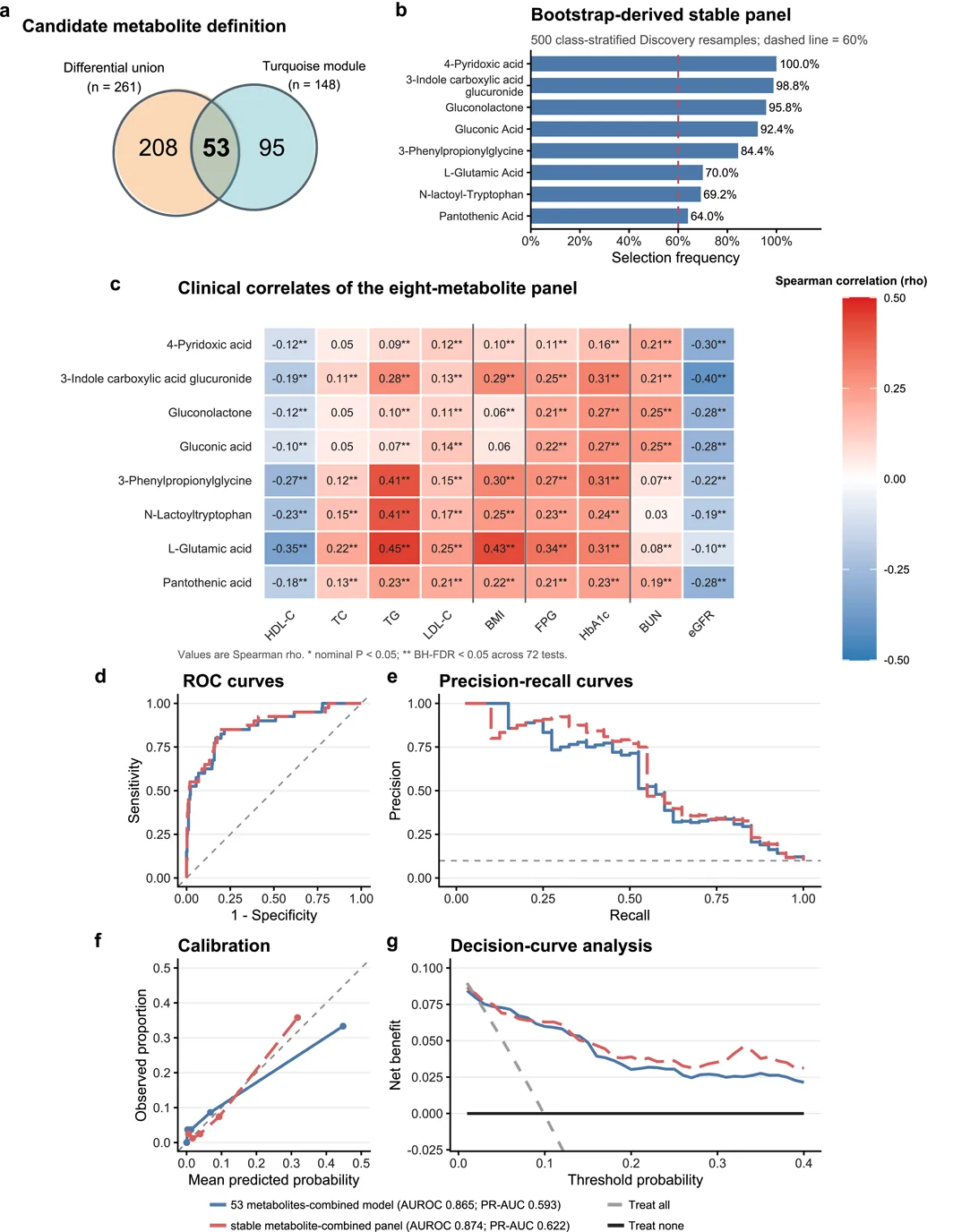
Integration of stage‐specific profiling, WGCNA and validation of advanced CKM classification. (a) Overlap between the 261‐metabolite differential union and the 148‐metabolite turquoise module, identifying 53 candidate metabolites. (b) Bootstrap selection frequencies of the eight metabolites retained in at least 60% of 500 class‐stratified Discovery‐cohort resamples. The dashed line indicates the 60% selection‐frequency threshold. (c) Spearman correlations between the stable eight‐metabolite panel and nine clinical markers. Cell values are Spearman correlation coefficients, *ρ*. One asterisk indicates nominal *p* < 0.05, and two asterisks indicate BH‐FDR < 0.05. (d) Receiver operating characteristic curves comparing the 53 metabolite‐combined model with the stable eight metabolite‐combined models in the Validation cohort. (e) Precision‐recall curves comparing the two penalised logistic models in the Validation cohort. (f) Calibration curves comparing observed and model‐predicted probabilities in the Validation cohort. The diagonal dashed line represents perfect calibration. (g) Decision‐curve analysis comparing the net clinical benefits of the two models across threshold probabilities in the Validation cohort. Grey and black reference lines represent the treat‐all and treat‐none strategies, respectively. BMI, body mass index; BUN, blood urea nitrogen; CKM, cardiovascular‐kidney‐metabolic; eGFR, estimated glomerular filtration rate; FPG, fasting plasma glucose; HbA1c, glycated haemoglobin; HDL‐C, high‐density lipoprotein cholesterol; LDL‐C, low‐density lipoprotein cholesterol; PR‐AUC, area under the precision‐recall curve; TC, total cholesterol; TG, triglycerides.

Within the turquoise module, γ‐glutamylvaline was the only metabolite meeting the prespecified hub criteria (Figure 3d; Supporting Information S2: Table S10). γ‐Glutamylvaline was also identified in stage 2 versus stage 0 and stage 3 versus stage 0 comparisons, providing consistent evidence from both metabolite‐level and network analyses. Functional enrichment of the turquoise module highlighted tryptophan metabolism, protein digestion and absorption, and arginine and proline metabolism (Supporting Information S1: Figure S9).

### 3.5 A Stable Eight‐Metabolite Panel Discriminates Advanced CKM in the Validation Cohort

The 261 unique stage‐associated metabolites were intersected with the 148 metabolites in the CKM‐associated turquoise module, yielding 53 candidate metabolites (Figure 4a; Supporting Information S2: Table S11). Bootstrap stability selection in the Discovery cohort retained eight metabolites in ≥ 60% of the resamples: 4‐pyridoxic acid, 3‐indole carboxylic acid glucuronide, gluconolactone, gluconic acid, 3‐phenylpropionylglycine, N‐lactoyltryptophan, L‐glutamic acid, and pantothenic acid (Figure 4b). All eight metabolites were positively correlated with TG, LDL‐C, FPG, and HbA1c and inversely correlated with HDL‐C and eGFR after BH correction; most were also positively associated with BMI and BUN (Figure 4c; Supporting Information S2: Table S12), linking the stable panel to multiple metabolic and kidney‐related dimensions of CKM.

In the validation cohort, the model combining age, sex and 53 candidate metabolites achieved an AUROC of 0.865 (95% CI, 0.794–0.923) and a PR‐AUC of 0.593 (95% CI, 0.457–0.723) for the discrimination between advanced and early CKM (Figure 4d,e). At the prespecified Discovery‐derived threshold, it yielded a sensitivity of 80.0%, specificity of 83.0%, balanced accuracy of 81.5%, and Brier score of 0.0629 (95% CI, 0.0504–0.0763) (Supporting Information S2: Table S13).

We next assessed the simplified model incorporating age, sex, and the further selected metabolites. Eight metabolites were retained based on non‐zero coefficients in ≥ 60% of bootstrap resamples. Despite the reduction in the number of predictors, the model incorporating age, sex, and eight metabolites showed comparable or slightly improved performance with an AUROC of 0.874 (95% CI, 0.802–0.931) and PR‐AUC of 0.622 (95% CI, 0.480–0.759). At the same threshold, sensitivity was 85.0%, specificity was 81.1%, and balanced accuracy was 83.0%, with a Brier score of 0.0619 (95% CI, 0.0536–0.0708). Compared with the 53 metabolites‐combined model, the 8 metabolite‐combined model showed a modest increase in AUROC (ΔAUROC = 0.009, 95% CI, 0.001–0.017; DeLong *p* = 0.032), whereas differences in PR‐AUC (ΔPR‐AUC = 0.029, 95% CI, −0.008 to 0.076) and Brier score (ΔBrier = −0.0011, 95% CI, −0.0066 to 0.0039) were not statistically significant. These findings indicate that feature reduction from 53 to 8 metabolites largely preserved predictive performance while improving model parsimony.

Calibration performance was broadly acceptable for both models. The 53 metabolites‐combined model showed a calibration intercept of −0.141 (95% CI, −0.423 to 0.136) and slope of 0.685 (95% CI, 0.496–0.969), whereas the 8 metabolite‐combined model showed an intercept of 0.062 (95% CI, −0.091 to 0.205) and slope of 1.231 (95% CI, 0.897–1.750) (Figure 4f,g; Supporting Information S2: Table S13). Calibration curves demonstrated similar agreement between predicted and observed risks, and decision‐curve analysis showed comparable net benefit across clinically relevant thresholds, with a modest advantage for the eight‐metabolite model.

Secondary machine‐learning algorithms demonstrated generally consistent discrimination. For the 8 metabolite‐combined model, AUROCs were 0.870 (95% CI, 0.798–0.925) for random forest, 0.845 (95% CI, 0.773–0.903) for XGBoost, and 0.825 (95% CI, 0.746–0.889) for radial‐basis SVM, compared with 0.874 (95% CI, 0.802–0.931) for penalised logistic regression. Penalised logistic regression showed the most balanced performance across discrimination, precision–recall characteristics, calibration, and model simplicity (Supporting Information S2: Table S13).

Overall, the stable metabolite‐combined panel retained the predictive information of the 53 metabolites‐combined model while substantially reducing the model complexity, supporting a compact molecular signature for advanced CKM classification.

### 3.6 Stratified and Sensitivity Analyses Support the Robustness of the Principal Findings

Sensitivity analyses supported the robustness of the stage‐associated metabolic profiles. After excluding participants with uncertain CKD classification, 82.4%–100% of primary metabolite sets were retained across comparisons, except for stage 4 versus stage 3 (67.9%), with 100% directional concordance among overlapping metabolites. Exclusion of stage 4 participants had minimal impact on the evaluable stage 0–3 comparisons (Supporting Information S2: Tables S14 and S15). No metabolite‐by‐sex or metabolite‐by‐age interaction remained significant after BH correction (Supporting Information S2: Table S16).

## 4 Discussion

In this cross‐sectional metabolomic study of 1374 participants from the China Multi‐Ethnic Cohort, we identified substantial molecular heterogeneity across the clinically defined CKM spectrum. Stage 1 was characterised primarily by lower lipid‐ and bile acid‐related metabolites, whereas stage 2 showed broader alterations involving monoacylglycerols, fatty acids, γ‐glutamyl peptides, and N‐acyl/lactoyl amino acids. Stage 3 was further distinguished by carbohydrate, aromatic amino‐acid, secondary bile acid, host–microbial, and renal‐handling signals, while metabolic separation between stages 3 and 4 was comparatively weak. Network analysis identified a CKM‐stage‐associated turquoise module, with γ‐glutamylvaline as a hub metabolite. Finally, machine learning reduced 53 candidate metabolites to a stable eight‐metabolite panel that, combined with age and sex, discriminated advanced CKM with an AUROC of 0.874 without incorporating conventional CKM‐defining variables.

Our findings extend the emerging metabolomic evidence that CKM represents a biologically heterogeneous spectrum rather than a single metabolic trajectory. A recent plasma metabolomic study identified distinct metabolic subtypes associated with CKM severity and substantial heterogeneity within high‐risk CKM [13]. By incorporating both stage 0–4 comparisons and adjacent‐stage contrasts, our study provides additional resolution regarding when metabolic remodelling occurs along CKM progression.

The earliest metabolic difference was observed at stage 1, where differential metabolites were predominantly decreased and enriched for lysophospholipids, oxidised fatty‐acid derivatives, and bile acid‐related metabolites. Lower circulating lysophosphatidylcholines have been reported in obesity and type 2 diabetes, while bile acids regulate glucose, lipid, and energy metabolism through receptors including FXR and TGR5 [33, 34]. These findings suggest that early CKM may involve subtle disturbances in lipid handling and bile acid homoeostasis before overt cardiorenal complications emerge.

A broader metabolic transition emerged at stage 2. The elevation of monoacylglycerols, fatty acids, γ‐glutamyl peptides, and N‐acyl/lactoyl amino acids, together with enrichment of lipid, redox, and amino‐acid pathways, suggests coordinated metabolic remodelling rather than isolated abnormalities. Circulating monoacylglycerols have previously been associated with visceral adiposity and hepatic triglyceride accumulation [35, 36], supporting their relevance to CKM‐related metabolic dysfunction. Among these alterations, γ‐glutamylvaline was particularly notable because it was identified both as a differential metabolite and as the hub metabolite of the CKM‐associated turquoise module. Previous studies have linked γ‐glutamyl peptides with metabolic syndrome, obesity, insulin resistance, and altered glucose–lipid metabolism [37, 38, 39]. Although these findings support γ‐glutamylvaline as a marker of coordinated metabolic disturbance, its causal role in CKM progression requires further investigation.

The stage 3 profile suggested a further qualitative shift towards carbohydrate, aromatic amino‐acid, host–microbial, and renal‐handling pathways. Phenylacetylglutamine and p‐cresol sulphate integrate microbial metabolism with host conjugation and renal elimination [40], and their circulating levels may reflect both microbial activity and impaired clearance in the setting of kidney dysfunction [41, 42]. This interpretation is particularly relevant for 4‐pyridoxic acid, which was retained in the stable metabolite panel. As an endogenous substrate of renal organic anion transporters OAT1 and OAT3, circulating 4‐pyridoxic acid reflects tubular secretory handling beyond conventional filtration markers [43, 44]. In the CRIC study, reduced pyridoxic acid clearance was associated with CKD progression independently of eGFR and albuminuria [45]. Accordingly, altered 4‐pyridoxic acid handling may reflect proximal tubular secretory function that is not fully captured by glomerular filtration. Plasma concentration alone, however, cannot distinguish altered production from reduced clearance.

Interestingly, stage 4 did not represent a simple extension of the stage 3 metabolic profile. Although stage 4 remained distinct from stage 0, the stage 4 versus stage 3 comparison showed limited metabolic separation, with predominantly lower metabolite concentrations. This finding may reflect both limited sample size and clinical heterogeneity among individuals with established cardiovascular disease, including differences in disease duration, treatment exposure, and underlying phenotypes. Thus, CKM severity may not correspond to a linear metabolic gradient.

A further contribution of this study is the identification of a parsimonious metabolic signature for advanced CKM. Accurate identification of individuals with advanced CKM remains an important clinical challenge, as these individuals carry substantially elevated risks of cardiovascular and kidney complications and may benefit from intensified monitoring and preventive strategies. The American Heart Association CKM framework emphasises stage‐based risk assessment but also highlights the need for additional biomarkers to refine risk stratification and biological characterisation.

Our eight‐metabolite panel provides a potential molecular complement to conventional CKM assessment. The model achieved an AUROC of 0.874 in the validation cohort despite excluding BMI, blood pressure, glycaemic indices, lipid measures, eGFR, and cardiovascular variables used for CKM stage assignment. Combining routinely available age and sex with a small metabolite panel enabled classification of advanced CKM without directly incorporating the clinical features defining the current staging system. Thus, the panel may capture biological heterogeneity not fully represented by conventional criteria. Given the potential molecular heterogeneity among individuals within the same CKM stage, the eight‐metabolite signature may serve as a candidate molecular phenotype for biological stratification and targeted validation.

The translational potential of this panel is further supported by its biological coherence. The selected metabolites represent multiple CKM‐relevant processes, including renal tubular handling, amino‐acid and nitrogen metabolism, carbohydrate metabolism, vitamin/cofactor metabolism, and host–microbial co‐metabolism. Several components of the panel have biological support from previous studies. Gluconic acid has been associated with CKD severity and kidney‐function decline [46], whereas glutamate metabolism links amino‐acid, nitrogen, and energy metabolism with metabolic disease [47]. Pantothenate metabolism, through co‐enzyme A biosynthesis, is closely connected with fatty‐acid oxidation and energy metabolism and has been implicated in diabetic kidney disease [48]. In addition, 3‐phenylpropionylglycine reflects gut microbial metabolism and PPAR‐related signalling [49], while altered tryptophan metabolism involving indole derivatives has been reported in CKD [50]. However, the biological relevance of several metabolites, including gluconolactone and N‐lactoyltryptophan, requires further validation. Future studies should determine whether standardised quantification of these metabolites improves CKM risk stratification beyond current clinical measures and whether the signature predicts clinically meaningful outcomes, including cardiovascular events, kidney‐function decline, and CKM‐stage transitions.

However, several limitations should be considered. First, the cross‐sectional design prevents assessment of temporal relationships or causal mechanisms, and external validation is required before clinical implementation. Second, CKM staging lacked several detailed measurements, including UACR, repeated kidney‐function assessments, cardiac biomarkers, imaging‐based cardiovascular phenotyping, and information on atrial fibrillation or peripheral artery disease, which may have resulted in stage misclassification. Third, residual confounding from medication use, diet, fasting status, environmental exposures, and renal clearance remains possible despite adjustment for major covariates. Finally, the limited number of advanced CKM cases may have affected model stability and precision.

## 5 Conclusion

In conclusion, CKM stages exhibit distinct and non‐linear metabolic signatures. Early CKM is characterised by lipid and bile acid alterations, whereas later stages involve broader disturbances in carbohydrate metabolism, renal handling, and host–microbial pathways. A stable eight‐metabolite signature captured multiple dimensions of CKM biology and maintained discrimination for advanced CKM without relying on conventional CKM‐defining variables. Although further validation is required, this approach provides a potential framework for molecular phenotyping and future refinement of CKM risk assessment.

## Author Contributions

**Xuemei Gong:** writing – original draft, writing – review and editing, methodology, visualization, software, investigation, validation, formal analysis, conceptualization. **Chunyang Li:** writing – review and editing, methodology, investigation, data curation, conceptualization. **Jing Chen:** investigation, data curation, conceptualization. **Yujiao Wang:** investigation, data curation, conceptualization. **Wenge Tang:** investigation, resources. **Xuehui Zhang:** resources, investigation. **Jianzhong Yin:** investigation, supervision, resources, project administration. **Xing Zhao:** supervision, resources, funding acquisition, project administration. **Haopeng Yu:** writing – review and editing, validation, supervision, resources, methodology, conceptualization. **Ping Fu:** writing – review and editing, validation, supervision, methodology, investigation, conceptualization. **Xiaoxi Zeng:** writing – review and editing, funding acquisition, visualization, supervision, software, resources, methodology, investigation, data curation, conceptualization. All authors approved the final version of the manuscript. Xiaoxi Zeng is the guarantor of this work and, as such, had full access to all the data in the study and takes responsibility for the integrity of the data and the accuracy of the data analysis.

## Funding

This work was supported by the General Program of the National Natural Science Foundation of China (Grant 72574155), and the National Key R&D Program of China (Grant 2017YFC0907300).

## Ethics Statement

The study was approved by the Sichuan University Medical Ethical Review Board and local ethics committee at each participating site.

## Consent

All participants provided informed consent for their data and biological samples to be used in approved scientific research.

## Conflicts of Interest

The authors declare no conflicts of interest.

## Supporting information


Supporting Information S1

Supporting Information S2

## Data Availability

The data that support the findings of this study are available on request from the corresponding author. The data are not publicly available due to privacy or ethical restrictions.
