# Supplementary material for: Metabolomic Profiling Delineates Stage‐Associated Metabolic Remodelling in Cardiovascular‐Kidney‐Metabolic Syndrome
Source: Diabetes Metab Res Rev. 2026 Sep 24;42(6):e70230. doi: 10.1002/dmrr.70230 (PMC13602901; doi:10.1002/dmrr.70230)
Supplement: Supplementary file 1 [file DMRR-42-e70230-s001.docx]

**Figure S1. Study flowchart.**

**Figure S2. Clinical correlations between differential metabolites and clinical features for CKM Stage 1 versus Stage 0. Colors indicate Spearman correlation coefficients, with blue and red denoting negative and positive correlations, respectively; darker colors indicate stronger absolute correlations. *, P < 0.05; **, BH-adjusted P < 0.05.**

**Figure S3. Clinical correlations between differential metabolites and clinical features for CKM Stage 2 versus Stage 0. Colors indicate Spearman correlation coefficients, with blue and red denoting negative and positive correlations, respectively; darker colors indicate stronger absolute correlations. *, P < 0.05; **, BH-adjusted P < 0.05.**

**Figure S4. Clinical correlations between differential metabolites and clinical features for CKM Stage 2 versus Stage 1. Colors indicate Spearman correlation coefficients, with blue and red denoting negative and positive correlations, respectively; darker colors indicate stronger absolute correlations. *, P < 0.05; **, BH-adjusted P < 0.05.**

**Figure S5. Clinical correlations between differential metabolites and clinical features for CKM Stage 3 versus Stage 0. Colors indicate Spearman correlation coefficients, with blue and red denoting negative and positive correlations, respectively; darker colors indicate stronger absolute correlations. *, P < 0.05; **, BH-adjusted P < 0.05.**

**Figure S6. Clinical correlations between differential metabolites and clinical features for CKM Stage 3 versus Stage 2. Colors indicate Spearman correlation coefficients, with blue and red denoting negative and positive correlations, respectively; darker colors indicate stronger absolute correlations. *, P < 0.05; **, BH-adjusted P < 0.05.**

**Figure S7. Clinical correlations between differential metabolites and clinical features for CKM Stage 4 versus Stage 0. Colors indicate Spearman correlation coefficients, with blue and red denoting negative and positive correlations, respectively; darker colors indicate stronger absolute correlations. *, P < 0.05; **, BH-adjusted P < 0.05.**

**Figure S8. Clinical correlations between differential metabolites and clinical features for CKM Stage 4 versus Stage 3. Colors indicate Spearman correlation coefficients, with blue and red denoting negative and positive correlations, respectively; darker colors indicate stronger absolute correlations. *, P < 0.05; **, BH-adjusted P < 0.05.**

**Figure S9. Functional pathway enrichment analysis of the turquoise module. The x-axis indicates pathway impact, and the y-axis indicates statistical significance.**
